# Impaired Bone Density and Quality in Type 1 Diabetes Mellitus: Prevalence and Key Clinical Correlations

**DOI:** 10.3390/jcm15031292

**Published:** 2026-02-06

**Authors:** Simona Zaccaria, Isabella Nardone, Sium Wolde Sellasie, Laura Giurato, Chiara Pecchioli, Pasquale Di Perna, Luigi Uccioli

**Affiliations:** 1 Division of Endocrinology and Diabetes, CTO Andrea Alesini Hospital, Department of Biomedicine and Prevention, University of Rome Tor Vergata, 00133 Rome, Italy; simona.zaccaria@students.uniroma2.eu (S.Z.); isabella.nardone@students.uniroma2.eu (I.N.); sium.woldesellasie@students.uniroma2.eu (S.W.S.); laura.giurato@aslroma2.it (L.G.); chiara.pecchioli@aslroma2.it (C.P.); pasquale.diperna@aslroma2.it (P.D.P.); 2 PhD School of Applied Medical-Surgical Sciences, University of Rome Tor Vergata, 00133 Rome, Italy

**Keywords:** type 1 diabetes mellitus, bone health, trabecular bone score, bone mineral density, microvascular complications

## Abstract

**Background:** Type 1 diabetes mellitus (T1DM) is associated with an increased risk of fragility fractures that cannot be fully explained by reduced bone mineral density (BMD), highlighting a potential role for bone quality impairment. The purpose of this study was to evaluate the prevalence of altered bone density and microarchitecture and to identify their main clinical correlates in adults with T1DM and seemingly adequate glycemic control at the time of assessment. **Methods:** Sixty-eight adults aged 18–69 years with T1DM attending a diabetes technology outpatient clinic were enrolled in this single-center, cross-sectional study. BMD at the lumbar spine, femoral neck, and total hip was assessed by dual-energy X-ray absorptiometry (DXA) and classified as reduced based on age and sex: Z-score < −2.0 SD for premenopausal women and men < 50 years, and T-score ≤ −2.5 SD for postmenopausal women and men ≥ 50 years. Bone microarchitecture was evaluated using trabecular bone score (TBS). Clinical, metabolic, and lifestyle variables were collected, including glycated hemoglobin (HbA1c; good control ≈ 7.0%/53 mmol/mol), diabetes duration, microvascular complications, and physical activity (PA) assessed by the International PA Questionnaire (IPAQ; moderate–high PA defined according to combined high and moderate IPAQ categories). **Results:** Reduced BMD was observed in 35.3% of patients and was associated with older age (*p* < 0.001), longer disease duration (*p* = 0.044), lower body mass index (*p* = 0.031), poorer glycemic control (*p* = 0.03), microvascular complications such as diabetic peripheral neuropathy (*p* = 0.028) and retinopathy (*p* = 0.045), and low PA (*p* = 0.012). Altered TBS was present in 45.6% of patients and was associated with older age (*p* < 0.001), longer diabetes duration (*p* = 0.011), higher HbA1c levels (*p* < 0.001), diabetic peripheral neuropathy (*p* = 0.002), retinopathy (*p* = 0.007), cardiovascular risk factors (dyslipidemia *p* = 0.002, hypertension *p* = 0.002), and low PA (*p* < 0.001). In multivariable analyses, older age and higher HbA1c were independently associated with reduced TBS, whereas moderate–high PA was associated with a lower likelihood of impaired bone microarchitecture. **Conclusions:** Impaired bone density and bone quality are highly prevalent in adults with T1DM and are frequently associated with longer disease duration, poorer metabolic control, and chronic complications. Our findings support the potential value of a combined assessment of BMD and TBS in fracture risk evaluation, together with strategies aimed at preventing diabetes-related complications and promoting healthy lifestyle behaviors.

## 1. Introduction

Type 1 diabetes mellitus (T1DM) is an autoimmune disease characterized by pancreatic β-cell destruction, leading to absolute insulin deficiency and chronic hyperglycemia [1]. This chronic condition contributes to both microvascular and macrovascular complications and seems to significantly affect bone health, although it remains debated whether skeletal fragility should be considered a comorbidity or a direct complication of the disease [2].

Patients with T1DM have an increased risk of fragility fractures, including vertebral and hip fractures, with hip fractures occurring 10–15 years earlier than in healthy controls [3,4]. Despite this, reductions in bone mineral density (BMD) are often modest, suggesting that BMD alone does not fully explain fracture risk. Indeed, some patients with fragility fractures may even have normal BMD [2,5,6,7,8]. Consequently, research attention has focused on bone quality as a key determinant of fracture risk [9,10].

Bone deterioration in T1DM is multifactorial. Chronic hyperglycemia, insulin deficiency, and low insulin-like growth factor-1 (IGF-1) levels contribute to impaired bone metabolism and a low bone turnover state, affecting skeletal microarchitecture [5,6,11]. Long-standing T1DM may also increase the risk of falls due to complications such as diabetic peripheral neuropathy (DPN), retinopathy, and muscle weakness, which can further compromise bone health [12,13,14]. Visual impairment and neuropathy may limit physical activity (PA), thereby promoting bone loss [13,14]. In addition, diabetic nephropathy, which is associated with negative calcium balance and reduced vitamin D levels, has been identified as an early indicator of osteopenia in T1DM [14]

Among these complications, DPN has emerged as a major independent factor contributing to impaired bone quality and reduced bone strength. DPN is strongly associated with lower cortical volumetric BMD, reduced cortical thickness, and increased cortical porosity, resulting in altered biomechanical properties and decreased estimated bone strength and stiffness [6].

Given the serious clinical consequences of diabetes-related bone fragility, preventive strategies should be integrated into patient management from an early stage, with optimal glycemic control remaining a cornerstone of care.

The associations demonstrated between T1DM, chronic complications, and bone tissue deterioration highlight the need for further investigation in well-controlled patients.

This study aimed to assess the prevalence of impaired bone density and quality in adults with type 1 diabetes and seemingly adequate metabolic control. We specifically investigated the associations of bone mineral density and trabecular bone score (TBS) with clinical factors, including age, disease duration, diabetic complications such as peripheral neuropathy and retinopathy, metabolic control, and physical activity. By focusing on these endpoints, we sought to identify key determinants of skeletal fragility in this population.

## 2. Materials and Methods

### 2.1. Case Selection

This was a single-center, cross-sectional study conducted between September 2022 and June 2023 at the Diabetes and Endocrinology Department of CTO Hospital, Rome, Italy.

A total of 68 patients with T1DM regularly attending the diabetes technology outpatient clinic were prospectively enrolled.

The criteria for inclusion were an age between 18 and 69 years and a diagnosis of T1DM from at least the last 5 years.

The criteria for exclusion were (a) presence of fractures due to major injuries, malignancies and other causes not related to bone fragility; (b) presence of severe or acute cardiovascular diseases that limit or contraindicate PA; (c) major lower amputations; (d) prior or actual presence of plant pressure ulcers; and (e) lack of consent to the processing of personal data.

A written informed consent was obtained from each patient to collect clinical data.

### 2.2. Protocol

Anthropometric and clinical variables were included: age, sex, menopausal status, body mass index (BMI, kg/m^2^), type of insulin therapy (insulin pump or basal–bolus therapy with continuous glucose monitoring—CGM), smoking and alcohol consumption habits, duration of disease (years), arterial hypertension, dyslipidemia, carotid atheromasia, autoimmune disease (Basedow–Graves’ disease, rheumatoid arthritis—AR, Celiac Disease) and microvascular complications (retinopathy, nephropathy, neuropathy), and PA levels. Laboratory data, collected at the time of the study visit, included glycated hemoglobin (HbA1c; % and mmol/mol), creatinine (mg/dL), estimated glomerular filtration rate (eGFR; mL/min), and albuminuria (mg/g).

According to international guidelines (ADA 2025; ISPAD 2022) [15,16], an HbA1c value around 7.0% (53 mmol/mol) is generally considered indicative of adequate glycemic control in adults with type 1 diabetes.

Diabetic retinopathy was diagnosed based on ophthalmologic examination. Hypertension was defined as a systolic blood pressure ≥ 140 mmHg, a diastolic blood pressure ≥ 90 mmHg, or ongoing antihypertensive therapy. Dyslipidemia was defined according to established criteria, including elevated LDL cholesterol levels above recommended targets for cardiovascular risk or the use of lipid-lowering medications, in line with 2019 ESC/EAS guidelines. Diabetic nephropathy was defined by the presence of albuminuria greater than 30 mg/g and/or eGFR < 60 mL/min/1.73 m^2^, confirmed on at least two measurements over a 3–6-month period.

DPN was investigated by evaluating the presence of symptoms through the Michigan Neuropathy Screening Instrument questionnaire [17] and signs using the Diabetic Neuropathic Index [18]; furthermore, electroneurography was conducted, comprehending the study of nerve conduction at the sural, motor peroneal and tibial nerves of the lower extremities.

Patients were classified according to the Toronto criteria as having absent, probable, possible, or confirmed diabetic peripheral neuropathy. For statistical analyses, “absent” and “probable” cases were combined into one group, while “possible” and “confirmed” cases were combined into another group.

PA levels were assessed using the International Physical Activity Questionnaire (IPAQ). Participants were initially classified into three categories [19]:

- High PA: engaging in vigorous-intensity activity for at least 1500 MET minutes per week on three or more days, or accumulating at least 3000 MET minutes per week through any combination of walking, moderate, or vigorous activities on seven or more days;
- Moderate PA: engaging in 20 min per day of vigorous activity on three or more days, 30 min per day of moderate activity or walking on five or more days, or accumulating at least 600 MET minutes per week through walking, moderate, or vigorous activities on five or more days;
- Low PA: not meeting the criteria for high or moderate PA.

For the purposes of statistical analysis, the high and moderate PA groups were combined into a single “moderate-high PA” category, while participants who did not meet these criteria were classified as low PA.

Data related to bone health were assessed in the same centre by experienced operators using Dual-energy X-ray Absorptiometry technique, with an Hologic Discovery densitometer [20] and included BMD (g/cm^2^) in the lumbar spine, femoral neck, and total hip. To ensure DXA scan quality, daily phantom scans were performed before clinical activities.

In accordance with international recommendations, different criteria were applied based on age and menopausal status. In premenopausal women and men under 50 years of age, reduced BMD was defined as a Z-score < −2.0 SD (“low BMD for age”). In postmenopausal women and men aged 50 years or older, reduced BMD was defined as a T-score ≤ −2.5 SD, consistent with densitometric osteoporosis.

For the purposes of analysis, participants were classified as having normal or reduced BMD.

Bone microarchitecture quality was assessed by Trabecular Bone Score, derived from DXA scans of the lumbar spine using TBS iNsight Imaging Software (version 3.0.2.0; Medimaps Group, Geneva, Switzerland) [21,22].

A TBS value greater than 1.350 was considered indicative of a normal bone microarchitecture, values between 1.200 and 1.350 indicated a partially degraded microarchitecture, and values ≤ 1.200 denoted a severely degraded microarchitecture [23].

In the lumbar scan, vertebrae showing artifacts due to arthrosis or vertebral collapse were excluded from the BMD and TBS calculation.

Anamnestic data were collected about fragility fractures, defined as spontaneous fractures or fractures after a minor injury or, in the case of asymptomatic vertebrate collapse, identified during radiological investigations. Fragility fractures may involve the spinal cord, hip or other anatomical sites.

### 2.3. Statistical Analyses

Data were analysed using Jamovi Version 2.3.28 [24]. A *p* value  <  0.05 was considered statistically significant. Baseline characteristics were reported as percentages for categorical variables, as mean ± standard deviation (SD) for normally distributed continuous variables, and as median with interquartile range (IQR) for non-normally distributed variables. Differences in quantitative variables across the two BMD and TBS categories were assessed using analysis of variance (ANOVA), while differences in qualitative variables were evaluated using the chi-square test.

Binomial logistic regression analysis was conducted to assess which factors were independently associated with the outcome.

## 3. Results

### 3.1. Study Population Characteristics

Sixty-eight patients with T1DM attending the diabetes technology outpatient clinic of CTO Hospital in Rome were enrolled in the study.

Thirty-three participants (48.5%) were female; among them, 18 (54.5%) were premenopausal and 15 (45.5%) postmenopausal.

The median age was 51.0 years (IQR 42.0–59.0), with a median disease duration of 22.5 years (IQR 15.0–34.0). Median BMI was 23.6 kg/m^2^ (IQR 21.9–26.6).

Most patients were treated with insulin pump therapy (*n* = 54, 79.4%), while 14 patients (20.6%) were on basal–bolus insulin therapy combined with continuous glucose monitoring. Glycemic control at the time of evaluation was overall adequate, with a median HbA1c of 7.2% (IQR 6.8–7.8). Microangiopathic complications were present in 26 patients, including retinopathy (*n* = 16), diabetic kidney disease (*n* = 7), and definite or probable DPN (*n* = 20).

### 3.2. Bone Mineral Density

As shown in Table 1, 44 patients (64.7%) had normal BMD, whereas 24 (35.3%) exhibited reduced BMD. Patients with reduced BMD were significantly older (*p* < 0.001; ε^2^ = 0.169), more frequently female (*p* = 0.027; Cramér’s V = 0.268), had a lower BMI (*p* = 0.031; ε^2^ = 0.07), and had a longer duration of diabetes (*p* = 0.044; ε^2^ = 0.06). Reduced BMD was also associated with a higher prevalence of diabetic retinopathy (*p* = 0.045; Cramér’s V = 0.243), DPN (*p* = 0.028; Cramér’s V = 0.266), and arterial hypertension (*p* = 0.016; Cramér’s V = 0.292), as well as with lower levels of PA (*p* = 0.012; Cramér’s V = 0.279).

Better metabolic control, reflected by lower HbA1c levels, and higher estimated glomerular filtration rate were associated with normal BMD (*p* = 0.030; ε^2^ = 0.06 and *p* = 0.050, ε^2^ = 0.06 respectively). All five patients who experienced fragility fractures belonged to the reduced BMD group (*p* = 0.002; Cramér’s V = 0.381).

No significant differences in BMD status were observed with respect to insulin therapy (*p* = 0.114), smoking habit (*p* = 0.232), alcohol consumption (*p* = 0.378), presence of nephropathy (*p* = 0.092), dyslipidemia (*p* = 0.065), carotid atheromasia (*p* = 0.066), creatinine (*p* = 0.419), or microalbuminuria (*p* = 0.645). Similarly, the prevalence of autoimmune diseases (Basedow–Graves’ disease, AR, celiac disease) did not differ between subjects with normal or reduced BMD (*p* = 0.131).

### 3.3. Trabecular Bone Score

Regarding trabecular bone score, 37 patients (54.4%) had normal values, while 31 (45.6%) showed partially degraded or degraded TBS (Table 2). Patients with reduced TBS were significantly older (*p* < 0.001; ε^2^ = 0.359) and had a longer disease duration (*p* = 0.011; ε^2^ = 0.100). Reduced TBS was more frequently observed in patients with diabetic retinopathy (*p* = 0.007; Cramér’s V = 0.328), DPN (*p* = 0.002; Cramér’s V = 0.381), carotid atheromasia (*p* = 0.024; Cramér’s V = 0.273), dyslipidemia (*p* = 0.002; Cramér’s V = 0.374), and arterial hypertension (*p* = 0.002; Cramér’s V = 0.367). These patients also exhibited poorer metabolic control and renal function, with higher HbA1c levels (*p* < 0.001; ε^2^ = 0.192) and lower eGFR (*p* < 0.001; ε^2^ = 0.214).

**Figure 1:**
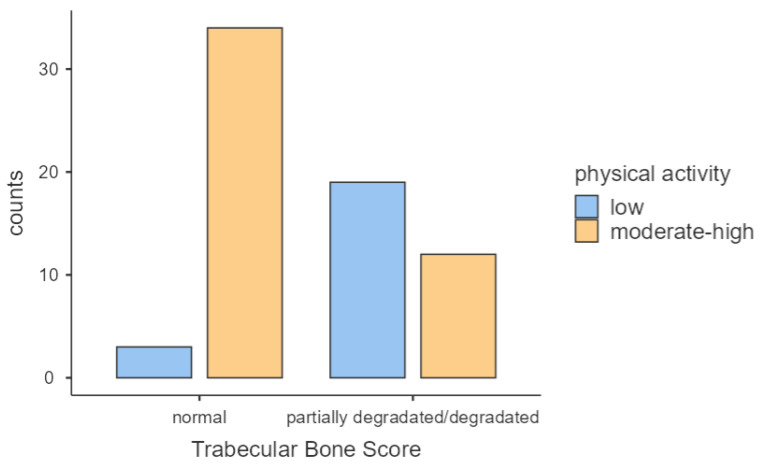
Relationship between trabecular bone score categories and physical activity levels.

Low physical activity was markedly more prevalent among patients with reduced TBS compared with those with normal TBS (61.3% vs. 8.1%, *p* < 0.001; Cramér’s V = 0.566; Figure 1).

Conversely, no significant differences were observed between TBS categories and sex (*p* = 0.054), insulin therapy (*p* = 0.818), BMI (*p* = 0.369), smoking status (0.641), alcohol consumption (*p* = 1.000), nephropathy (*p* = 0.400), serum creatinine levels (*p* = 0.627), microalbuminuria (*p* = 0.875), or presence of autoimmune diseases (*p* = 0.0069). Notably, all fragility fractures occurred in patients with reduced TBS (*p* = 0.016; Cramér’s V = 0.308).

### 3.4. Fragility Fractures

The fragility fracture group showed clear evidence of compromised bone quality compared with patients without fractures. Specifically, the median trabecular bone score in patients with fragility fractures was 1.21 (IQR: 1.21–1.25), compared with 1.37 (IQR: 1.29–1.43) in those without fractures (*p* = 0.008).

Patients with fragility fractures also exhibited significantly lower bone mineral density at both the lumbar spine (*p* = 0.014) and total hip (*p* = 0.015), whereas femoral neck BMD showed a non-significant trend toward lower values (*p* = 0.155).

Regarding comorbidities and clinical risk factors, patients with fragility fractures were significantly older (*p* < 0.001), had a longer duration of diabetes (*p* = 0.035), and showed a higher prevalence of definite or probable diabetic peripheral neuropathy (*p* = 0.024) and diabetic retinopathy (*p* = 0.009) compared with patients without fractures.

In terms of PA, all patients who experienced fragility fractures were classified as having low physical activity levels (*p* = 0.003).

No statistically significant differences were observed between the two groups with respect to sex (*p* = 0.191), smoking status (*p* = 0.191), alcohol consumption (*p* = 0.520), glycated hemoglobin levels at the time of evaluation (*p* = 0.088), or BMI (*p* = 0.118).

### 3.5. Multivariable Analyses

In multivariable logistic regression analysis, higher HbA1c levels were independently associated with an increased likelihood of reduced BMD (OR 3.04, 95% CI 1.11–8.33, *p* = 0.031), whereas higher BMI was associated with a decreased likelihood (OR 0.73, 95% CI 0.59–0.91, *p* = 0.005). Age, disease duration, sex, and PA were not independently associated with reduced BMD.

Older age (OR 1.15, 95% CI 1.04–1.26, *p* = 0.004) and higher HbA1c (OR 5.42, 95% CI 1.35–21.69, *p* = 0.017) were independently associated with reduced TBS. Conversely, PA exerted a protective effect, reducing the odds of low TBS by approximately 85% (OR 0.15, 95% CI 0.03–0.88, *p* = 0.035). Disease duration, BMI, and sex were not independently associated with TBS.

## 4. Discussion

In this cross-sectional study, we investigated BMD and TBS as complementary indicators of skeletal health in adults with T1DM with apparently adequate glycemic control at the time of assessment. Our findings confirm the presence of bone involvement in T1DM and highlight the multi-factorial nature of diabetes-related skeletal fragility.

As expected, older age and longer disease duration were associated with reduced BMD. Among modifiable risk factors, better glycemic control and higher levels of physical activity were significantly associated with more favorable indices of bone quantity and quality.

Ageing and menopausal status are well-established determinants of bone loss in the general population and are particularly relevant in patients with type 1 diabetes mellitus. Advancing age is associated with progressive reductions in bone mineral density and deterioration of bone microarchitecture, while menopause accelerates bone loss through estrogen deficiency, leading to increased bone resorption and reduced bone strength [25,26].

In the context of T1DM, these physiological processes may interact with diabetes-specific factors, including chronic hyperglycemia, insulin deficiency, and long-term disease burden, further compromising skeletal health.

In our cohort, patients with reduced BMD and altered TBS were older and more frequently female, reflecting the expected influence of ageing and menopausal status on bone outcomes.

However, the persistence of significant associations between bone impairment and metabolic control, chronic complications, and physical activity in multivariable analyses suggests that skeletal fragility in T1DM cannot be explained by ageing or menopause alone, supporting the presence of disease-specific mechanisms affecting bone quality.

Current recommendations advocate bone health monitoring in patients with T1DM using dual-energy X-ray absorptiometry, starting at 50 years of age or earlier in the presence of additional risk factors, such as micro- or macrovascular complications, suboptimal glycemic control, family history of fragility fractures, or coexisting conditions such as celiac disease [23].

Our data further support the need for comprehensive skeletal assessment in this population, including evaluation of bone quality through TBS, particularly in older individuals with longer disease duration. Earlier assessment may also be warranted in patients with poor metabolic control or low levels of physical activity.

Although the number of fragility fractures observed was limited, all fractured individuals exhibited concomitant reductions in both BMD and TBS. Notably, fracture occurrence clustered with specific clinical characteristics: fractured patients were older, had longer disease duration, and showed a higher prevalence of chronic microvascular complications, including diabetic peripheral neuropathy and retinopathy. This pattern suggests that cumulative disease burden and prolonged exposure to diabetes-related metabolic and vascular alterations may synergistically contribute to progressive deterioration of both bone mass and microarchitecture.

From a pathophysiological perspective, bone loss in T1DM may already be present at disease onset and appears to progress more rapidly during the early years of diabetes, before stabilizing or partially normalizing over time. However, the development of microvascular complications, particularly when occurring before the attainment of peak bone mass or during phases of ongoing bone loss, may contribute to a later deterioration of skeletal integrity, involving both trabecular and cortical compartments [27].

Supporting this hypothesis, previous studies have demonstrated a link between microvascular disease and impaired bone microarchitecture. Vilaka et al. [28] reported increased cortical porosity in T1DM patients with DPN, while other cross-sectional studies have demonstrated reduced cortical thickness and decreased estimated bone strength in individuals with DPN [6].

TBS emerged as a particularly informative marker of bone quality. In our cohort, altered TBS was independently associated with older age and poorer glycemic control, as reflected by higher HbA1c levels, while regular moderate–high PA associated with more favourable bone microarchitecture. These findings are consistent with previous literature identifying TBS as a robust predictor of fragility fractures in both the general population and individuals with T1DM [29].

Moreover, reductions in BMD were generally modest and insufficient to fully explain fracture risk, consistent with prior evidence. Vestergaard [8] showed that, when fracture risk is estimated based on BMD alone, individuals with type 1 diabetes have only a 1.4-fold higher risk compared with non-diabetic controls, suggesting that reliance on BMD may underestimate the true fracture risk.

This conceptual framework supports our methodological choice of integrating TBS with BMD to better capture diabetes-related skeletal fragility.

While BMI was positively associated with BMD, it showed no significant relationship with TBS, underscoring the ability of TBS to capture qualitative skeletal alterations that are not reflected by bone density alone.

The strong association between low PA and reduced TBS further highlights the role of lifestyle factors in the maintenance of bone microarchitecture in T1DM.

Given that physical inactivity is a well-established contributor to the development of DPN [30], the emerging evidence linking inactivity to impaired bone microarchitecture provides additional support for lifestyle interventions as a cornerstone of diabetes management.

Physical activity was associated with more favorable bone microarchitecture and may potentially reduce the risk of diabetic peripheral neuropathy and fragility fractures, highlighting its possible protective role.

Encouraging regular moderate-vigorous PA, in accordance with international guidelines, may therefore play a dual protective role by preserving bone quality and reducing the risk of both DPN and fragility fractures.

From a clinical perspective, maintaining optimal bone health in patients with T1DM requires the implementation of general preventive strategies, including adequate calcium intake, vitamin D supplementation when indicated, and the promotion of regular PA [31] These measures should be integrated into routine diabetes care, even in patients with apparently good metabolic control.

The main strengths of this study include the use of validated and reproducible methods for TBS assessment, gold-standard criteria for the diagnosis of diabetic peripheral neuropathy, and standardized tools for the evaluation of physical activity levels.

The primary limitation is the relatively small sample size (*N* = 68); however, this is mitigated by the high post hoc statistical power (94.4%) observed for the association between PA and TBS, supporting the robustness of our findings.

Several methodological limitations should be acknowledged. The cross-sectional design prevents assessment of temporal or causal relationships between glycemic control, diabetes-related complications, lifestyle factors, and skeletal outcomes, all of which may exert time-dependent effects on bone health. Glycemic control was assessed only at the time of the study visit, and no longitudinal HbA1c or continuous glucose monitoring data were available; therefore, the study reflects seemingly adequate glycemic control at the time of assessment, rather than sustained long-term metabolic control.

Finally, although TBS is a validated and clinically useful tool, it provides an indirect estimate of trabecular microarchitecture from two-dimensional DXA images and does not capture cortical bone parameters or true microstructural alterations.

Despite these limitations, the study provides valuable insights into the determinants of skeletal fragility in adults with T1DM and highlights the potential benefits of modifiable factors, particularly regular physical activity.

## 5. Conclusions

In conclusion, advanced age, longer disease duration, and poorer glycemic control are key determinants of impaired bone quality and quantity in adults with T1DM.

Physical activity represents an important modifiable factor, associated with the preservation of bone microarchitecture and potentially contributing to a reduced risk of diabetic peripheral neuropathy and fragility fractures.

Fracture risk assessment, prevention of diabetes-related complications, and promotion of healthy lifestyle behaviours should therefore be considered integral components of routine diabetes care to optimize skeletal health in this population. The combined assessment of bone mineral density and trabecular bone score provides complementary information on bone quantity and quality and may help identify individuals at higher fracture risk.

The cross-sectional design, relatively small sample size, single time-point assessment of glycemic control, and indirect nature of TBS as a measure of trabecular microarchitecture should be considered when interpreting these findings. Nevertheless, the present study offers meaningful insights into the determinants of skeletal fragility in T1DM and highlights the potential benefit of lifestyle interventions, particularly regular physical activity. Larger longitudinal and interventional studies are warranted to further define preventive strategies for skeletal complications in T1DM, with particular attention to long-term glycemic exposure, microvascular complications, and direct assessment of bone microarchitecture.

## Figures and Tables

**Table 1:** Anthropometric, Clinical, Metabolic, and Bone Characteristics of Type 1 Diabetes Mellitus Patients (*N* = 68), stratified by Bone Mineral Density status. Continuous variables are presented as median (interquartile range, IQR) and were compared using the Kruskal–Wallis test. Categorical variables are expressed as number and percentage [*n* (%)] and were compared using the chi-square test or Fisher’s exact test, as appropriate. *p*-values in the final column indicate the statistical significance of differences between patients with normal versus reduced BMD.

Variables	Bone Mineral Density		*p* Value
	**Normal 44 (64.7%)**	**Reduced 24 (35.3%)**	
Age (years)	47.5 (41.0–52.0)	64.5 (50.3–69.0)	**<0.001**
Sex *n* (%)	Male 27 (61.4)	8 (33.3)	**0.027**
	Female 17 (38.4)	16 (66.7)	
BMI (kg/m^2^)	24.2 (22.2–27.5)	22.3 (21.2–24.7)	**0.031**
Smoking *n* (%)	25 (56.8)	10 (41.7)	0.232
Alcohol *n* (%)	7 (15.9)	2 (8.0)	0.378
DM duration (years)	21.0 (14.0–29.8)	32.0 (19.5–39.3)	**0.044**
Therapy *n* (%)	Insulin pump 32 (72.7) Basal-bolus insulin + CGM 12 (27.3)	22 (91.7) 2 (8.3)	0.114
Retinopathy *n* (%)	7 (15.9)	9 (37.5)	**0.045**
Diabetic polyneuropathy certain/possible *n* (%)	9 (20.5)	11 (45.8)	**0.028**
Nephropathy *n* (%)	2 (4.5)	4 (16.7)	0.092
Carotid atheromasia *n* (%)	5 (11.4)	7 (29.2)	0.066
Dyslipidemia *n* (%)	25 (56.8)	19 (79.2)	0.065
Hypertension *n* (%)	11 (25.0)	13 (54.2)	**0.016**
HbA1c %(mmol/mol)	7.05 (53.6) (6.60–7.53) (48.6–58.8)	7.55 (59) (7.05–8.05 (53.6–64.5))	**0.030**
Creatinine (mg/dL)	0.87 (0.77–0.95)	0.84 (0.76–0.92)	0.419
eGFR (mL/min/1.73 m^2^)	103.0 (87.4–109)	88 (73.5–105)	**0.050**
Albuminuria (mg/g)	5 (5.0–8.07)	5 (5.0–10.2)	0.645
Physical activity *n* (%)	Low 9 (20.5)	12 (50.0)	
	Moderate–high 35 (79.5)	12 (50.0)	**0.012**
Autoimmune diseases *n* (%)	5 (11.4)	4 (16.7)	0.710
Fragility fracture *n* (%)	0 (0.0)	5 (20.8) (2 (40) hip, 3 (60) vertebral)	**0.002**

Body mass index (BMI), diabetes mellitus (DM), glycated hemoglobin (HbA1c), Estimated Glomerular Filtration Rate (eGFR). Bold values indicate statistically significant *p* values.

**Table 2:** Anthropometric, Clinical, Metabolic, and Bone Characteristics of Type 1 Diabetes Mellitus Patients (*N* = 68), stratified by Trabecular Bone Score (TBS). Continuous variables are presented as median (interquartile range, IQR) and were compared using the Kruskal–Wallis test. Categorical variables are expressed as number and percentage [*n* (%)] and were compared using the chi-square test or Fisher’s exact test, as appropriate. *p*-values in the final column indicate the statistical significance of differences between patients with normal versus partially degraded/degraded TBS.

Variables	Trabecular Bone Score		*p* Value
	**Normal 37 (54.4%)**	**Partially Degraded/Degraded 31 (45.6%)**	
Age (years)	46.0 (30.0–51.0)	59.0 (50.5–68.0)	**<0.001**
Sex *n* (%)	Male 23 (62.2)	12 (38.7)	0.054
	Female 14 (37.8)	19 (61.3)	
BMI (kg/m^2^)	23.8 (21.3–25.0)	23.5 (22.1–28.2)	0.369
Smoking *n* (%)	20 (54.1)	15 (48.4)	0.641
Alcohol *n* (%)	5 (13.5)	4 (12.9)	1.000
DM duration (years)	18.0 (11.0–27.0)	32.0 (20.5–38.5)	**0.011**
Therapy *n* (%)	Insulin pump 29 (78.4) basal-bolus insulin + CGM 8 (21.6)	25 (80.6) 6 (19.4)	0.818
Retinopathy *n* (%)	4 (10.8)	12 (38.7)	**0.007**
Diabetic polyneuropathy certain/possible *n* (%)	5 (13.5)	15 (48.4)	**0.002**
Nephropathy *n* (%)	2 (5.4)	4 (12.9)	0.400
Carotid atheromasia *n* (%)	3 (8.1)	9 (29.0)	**0.024**
Dyslipidemia *n* (%)	18 (48.6)	26 (82.9)	**0.002**
Hypertension *n* (%)	7 (18.9)	17 (54.8)	**0.002**
HbA1c %(mmol/mol)	6.90 (51.9) (6.5–7.5) (47.5–58.5)	7.6 (59.6) (7.2–8.0 (55.2–63.9))	**<0.001**
Creatinine (mg/dL)	0.86 (0.76–0.91)	0.84 (0.76–0.95)	0.627
eGFR (mL/min/1.73 m^2^)	105.0 (95–116)	87.4 (74–97.5)	**<0.001**
Albuminuria (mg/g)	5 (4.4–8.3)	5 (5.0–8.0)	0.875
Physical activity *n* (%)	Low 3 (8.1)	19 (61.3)	
	Moderate–high 34 (91.9)	12 (38.7)	**<0.001**
Autoimmune disease (Basedow, AR, Celiac Disease) *n* (%)	2 (5.4)	7 (22.6)	0.069
Fragility fracture *n* (%)	0 (0.0)	5 (16.1) (2 (40) hip, 3 (60) vertebral)	**0.016**

Body mass index (BMI), diabetes mellitus (DM), glycated hemoglobin (HbA1c), and Estimated Glomerular Filtration Rate (eGFR). Bold values indicate statistically significant *p* values.

## Data Availability

The original contributions presented in this study are included in the article. Further inquiries can be directed to the corresponding author.
